# Efficacy and safety of high-dose intramuscular vitamin D_2_ injection in type 2 diabetes mellitus with distal symmetric polyneuropathy combined with vitamin D insufficiency: study protocol for a multicenter, randomized, double-blinded, and placebo-controlled trial

**DOI:** 10.3389/fendo.2023.1202917

**Published:** 2023-07-07

**Authors:** Tao Chen, Xiaoyan Xing, Lihua Huang, Mei Tu, Xiaoli Lai, Shidi Wen, Jin Cai, Shenglong Lin, Youping Zheng, Yuehui Lin, Lijuan Xu, Yuwen Qiu, Lumin Qiu, Yuebo Xu, Peiwen Wu

**Affiliations:** ^1^ Department of Endocrinology, Clinical Research Center for Metabolic Diseases of Fujian Province, the First Affiliated Hospital, Fujian Medical University, Fuzhou, China; ^2^ Department of Endocrinology and Metabolism, Longyan First Affiliated Hospital of Fujian Medical University, Longyan, China; ^3^ Department of Endocrinology and Metabolism, China-Japan Friendship Hospital, Beijing, China; ^4^ Department of Tumor Radiotherapy, Longyan First Affiliated Hospital of Fujian Medical University, Longyan, China; ^5^ Department of Severe Liver Disease, Mengchao Hepatobiliary Hospital of Fujian Medical University, Fuzhou, China; ^6^ Department of Ultrasound, Longyan First Affiliated Hospital of Fujian Medical University, Longyan, China; ^7^ Department of Endocrinology and Metabolism, Longyan Traditional Chinese Medicine Affiliated Hospital of Fujian University of Traditional Chinese Medicine, Longyan, China; ^8^ Department of Endocrinology and Metabolism, Longyan Shanghang County Hospital, Longyan, China; ^9^ Department of Diabetes, Longyan Boai Hospital, Longyan, China

**Keywords:** type 2 diabetes mellitus, distal symmetric polyneuropathy, high-dose vitamin D supplementation, Michigan neuropathy screening instrument, randomized controlled trial

## Abstract

**Background:**

Distal symmetric polyneuropathy (DSPN) is the most common chronic complication of type 2 diabetes mellitus (T2DM). DSPN may lead to more serious complications, such as diabetic foot ulcer, amputation, and reduced life expectancy. Observational studies have suggested that vitamin D deficiency may be associated with the development of DSPN in T2DM. However, interventional studies have found that low-dose vitamin D supplementation does not significantly improve neuropathy in DSPN. This study aims to evaluate the efficacy and safety of intramuscular injection of high-dose vitamin D (HDVD) in T2DM with DSPN combined with vitamin D insufficiency.

**Methods and analysis:**

We will conduct a multicenter, randomized, double-blinded, and placebo-controlled trial in four large hospitals. All eligible participants will be randomly assigned to either the vitamin D_2_ supplement or placebo control group and injected intramuscularly monthly for 3 months. Additionally, anthropometric measurements and clinical data will be collected at baseline and 3 months. Adverse events will be collected at 1, 2, and 3 months. The primary outcome measure is the change in the mean Michigan Neuropathy Screening Instrument (MNSI) score at baseline and 3 months post-intervention. We will use the gold-standard liquid chromatography-tandem mass spectrometry method to distinguish between 25(OH)D_2_ and 25(OH)D_3_ levels. The MNSN score before the intervention will be used as a covariate to compare the changes between both groups before and after the intervention, and the analysis of covariance will be used to analyze the change in the MNSI score after HDVD supplementation.

**Discussion:**

Glycemic control alone does not prevent the progression of DSPN in T2DM. Some studies have suggested that vitamin D may improve DSPN; however, the exact dose, method, and duration of vitamin D supplementation are unknown. Additionally, neuropathy repair requires HDVD supplementation to sustain adequate vitamin D levels. This once-a-month intramuscular method avoids daily medication; therefore, compliance is high. This study will be the first randomized controlled trial in China to analyze the efficacy and safety of HDVD supplementation for patients with T2DM and DSPN and will provide new ideas for pharmacological research and clinical treatment of diabetic neuropathy.

**Clinical trial registration:**

https://www.chictr.org.cn/, identifier ChiCTR2200062266.

## 1 Introduction

According to the latest International Diabetes Federation statistics for 2021, the total number of people with diabetes worldwide is 536.6 million—and China accounts for a quarter (1). The prevalence of diabetes among adults in China is 12.8%, higher than the global average of 10.5%. Moreover, the number of people with diabetes in China is approximately 129.8 million, and those with type 2 diabetes mellitus (T2DM) account for > 90%. Therefore, T2DM is a serious public health problem in China (2). Additionally, the chronic complications caused by T2DM are a threat to national health. Notably, the complications involving peripheral nerves—peripheral neuropathy—can affect 75% of people with T2DM. Peripheral neuropathy occurs as early as in the pre-diabetic state of obesity, indicating its close relation to diabetes, obesity, and other metabolic diseases (3, 4).

Distal symmetrical polyneuropathy (DSPN) is the most representative manifestation of nerve damage in T2DM and is more common than other spinal nerves, cranial nerves, and autonomic neuropathy lesions (5). DSPN develops slowly, starting with small nerve fibers at the end of the limb and progressing to larger nerves, including sensory and motor nerves. The symmetrical onset of numbness and sensory abnormalities is the earliest clinical symptom in many patients with DSPNs. Moreover, approximately one-third of patients experience burning, pin and needle sensations, and pain. These painful symptoms are unbearable and lead to insomnia and depression, reducing patients’ quality of life (6, 7). Additionally, patients with DSPN are prone to complications, such as diabetic foot ulcers, due to a long-term lack of self-sensory protection, leading to serious complications, such as irreversible amputation and shortened life expectancy. Finally, as the disease progresses, people’s ability to work becomes severely affected, causing a serious economic burden and wastage of resources for individuals, families, and society; therefore, DSPN requires more attention (8, 9).

The clinical efficacy of DSPN treatment is poor. The Action to Control Cardiovascular Risk in Diabetes (ACCORD) study showed that glycemic control alone in T2DM does not reduce the incidence of DSPN, suggesting that hyperglycemia is not the only factor influencing the development of DSPN. Recently, clinicians have been looking for alternative treatments to improve DSPN (10). The etiology and pathogenesis of DSPN have not been elucidated; however, its development involves or is accelerated by abnormal insulin signaling pathways, microcirculatory disorders, non-enzymatic advanced glycosylation end product pathways, oxidative stress, and other factors leading to mitochondrial dysfunction, DNA damage, and apoptosis (9, 11).

Vitamin D is a fat-soluble ring-opening steroid discovered over 100 years ago and includes vitamin D_2_ (ergocalciferol) and D_3_ (cholecalciferol). Vitamin D_2_ is derived from foods, such as mushrooms, offal, egg yolks, and fatty sea fish. Vitamin D_3_ is converted from 7-dehydrocholesterol in the skin by ultraviolet light. Vitamins D_2_ or D_3_ require two hydroxylation processes, mediated by 25-hydroxylase in the liver and 1α-hydroxylase in the kidneys, to become active as 1,25-(OH)_2_D_2_ or 1,25-(OH)_2_D_3_, respectively. At present, activated vitamin D has gone beyond the traditional concept of vitamin and as it can play a hormone-like role, so 1,25-(OH)_2_D is also known as D hormone. Therefore, some scholars refer to the unactuated form of vitamin D as a D prohormone. Additionally, activated vitamin D is directly involved in gene regulation *via* the vitamin D receptor (VDR), which has several biological roles. Since vitamin D receptors are found in bones, kidneys, parathyroid glands, small intestine, nerve tissue, and pancreatic β-cell, vitamin D may have other biological roles in addition to its traditional role of regulating calcium, phosphorus, and bone metabolism. These roles include facilitating cell differentiation and proliferation, reducing superoxide, regulating lipid metabolism and inflammatory factors, reducing superoxide, promoting nerve growth factors, regulating immunity, reducing cytokine storms, and reducing COVID-2019 mortality (12–16).

Considering all these functions, vitamin D is important for health; however, its insufficiency is a widespread public health problem. Vitamin D nutritional status is determined by measuring serum 25-(OH)D levels—a sum of 25-(OH)D_2_ and 25-(OH)D_3_. The Endocrine Society recommends a 25-(OH)D level of 30 ng/mL (17); a patient is considered to have a vitamin D -insufficient state if this level is not reached (17–19). Studies have shown that vitamin D deficiency occurs in 74.7% of people with T2DM and affects people with prediabetes. Moreover, studies have shown that people with low vitamin D levels and prediabetes have a significantly higher risk of developing diabetes (20, 21).

A study by the National Health and Nutrition Examination Survey showed that the risk of developing DSPN increases by 2.59 times in people with vitamin D deficiency (22). Another study suggested that vitamin D deficiency may be an independent risk factor for DSPN (23). In an observational study, Seham et al. (24) found that vitamin D levels were significantly lower in patients and DSPN than in those with T2DM without DSPN and confirmed that 87.6% of those with DSPN had vitamin D deficiency, and the prevalence of vitamin D deficiency in T2DM without DSPN was only 45%.

However, the mechanisms by which vitamin D deficiency specifically affects DSPN are unknown. Vitamin D may improve glycemic control by promoting β-cell secretion and increasing insulin sensitivity. Additionally, vitamin D may reduce oxidative stress and prevent nerve damage (25). Animal studies have shown that vitamin D increases the synthesis of nerve growth factors in rats (26). Vitamin D is a potent inducer of neurotransmitters and can reduce nerve demyelination and improve axonal regeneration (27, 28).

A meta-analysis showed that vitamin D supplementation effectively reduced neuropathic pain and prevented further nerve damage (29). The association between vitamin D and pain may be due to nociceptive calcitonin gene-related peptide (CGRP)-positive neurons. These neurons have a distinct vitamin D phenotype, and their ligands and receptors are hormonally regulated. A study reported that vitamin D receptor expression in the growth cones and CGRP expression increased rapidly with vitamin D deficiency, indicating a possible connection between vitamin D and pain. Additionally, this study suggested the presence of lower pain tolerance in patients with vitamin D deficiency (30, 31).

Adequate vitamin D levels are not achieved with sunlight and food intake alone; therefore, additional supplements are required. Studies have shown that different doses of vitamin D supplementation may produce different results in DSPN treatment. A clinical study by Karonova et al. (32) showed that high-dose vitamin D (HDVD) supplementation in patients with DSPN and vitamin D deficiency achieved adequate status in all patients; however, only half of the low-dose group achieved adequate status. Moreover, the severity of neuropathy was accompanied by a significant reduction in inflammatory markers in the HDVD group; however, these markers were not altered in the low-dose group. Basit et al. (33) showed a significant decrease in total pain score and a reduction in positive symptoms in diabetic neuropathy patients treated with HDVD. Silva et al. (34) treated patients who had diabetic cardiac autonomic neuropathy with HDVD and showed significant improvement in parameters related to resting heart rate variability without adverse effects. Notably, neuropathy repair and symptom improvement are slow; therefore, we decided to use HDVD supplementation to repair neuropathy.

It is currently believed that vitamin D_2_ and D_3_ supplementation have the same effect of correcting vitamin D insufficiency, and it is believed that injection is better than oral supplementation (17, 35). Indeed, lower daily doses of vitamin D supplements require longer time to increase vitamin D levels in the body and are less likely to sustain sufficient levels. In addition, daily medication reduced compliance and executive function in patients, thereby reducing the number of people who reach sufficient state of vitamin D. Therefore, some scholars have proposed that the injection of HDVD supplementation as a method of administration with lower frequency and longer interval will be more conducive to obtain desired intervention outcomes (36, 37). Compared to vitamin D_3_, vitamin D_2_ has a lower binding affinity to vitamin D-binding proteins (VDBP). This means that taking vitamin D_2_ instead of vitamin D_3_ will result in higher levels of free vitamin D concentration in the blood, which will bind better to VDR (38, 39). Indeed, vitamin D_2_ has been used clinically for more than 50 years (39). Therefore, we use vitamin D_2_ injection as a strategy to correct vitamin D insufficiency.

The traditional nerve conduction test reflects only the lesions of large nerve fibers but not those of small fibers, such as fine myelinated A δ, unmyelinated C, and autonomic nerve fibers, which mediate nociceptive pain and temperature perception (5). Michigan Neuropathy Screening Instrument (MNSI) comprises a questionnaire and physical examination. The questionnaire contains 15 questions that address DSPN symptoms, with a maximum score of 13 points, and is completed by the patients. The physical examination is performed by the physician and includes ankle reflexes, vibration perception, foot ulceration, and feet appearance. Physical examination has a maximum score of 8 points for both feet. Multiple studies have demonstrated that MNSI is a reliable and effective tool for assessing DSPN. Additionally, compared with nerve conduction tests, MNSI avoids invasive examinations and the discomfort of electrical stimulation. Moreover, MNSI may avoid missing early small-fiber lesions in DSPN (40–44). Therefore, we will use the MNSI to assess the changes in neuropathy after HDVD supplementation.

The development of DSPN is relatively reversible in the early stage; however, it becomes a refractory nerve injury in the later stage, and the condition is persistent and recurrent. Correcting vitamin D deficiency helps prevent and treat DSPN. However, the specific dosage, long-term treatment effects, and related side effects should be confirmed in high-quality randomized controlled trials. Few clinical trials have investigated the effects of vitamin D supplementation on neuropathy in China’s population with DSPN and T2DM. Therefore, this study aims to explore the efficacy of HDVD supplementation in treating DSPN in T2DM, assess its safety, and collect information on adverse reactions.

## 2 Methods and analysis

### 2.1 Study design

**Figure 1:**
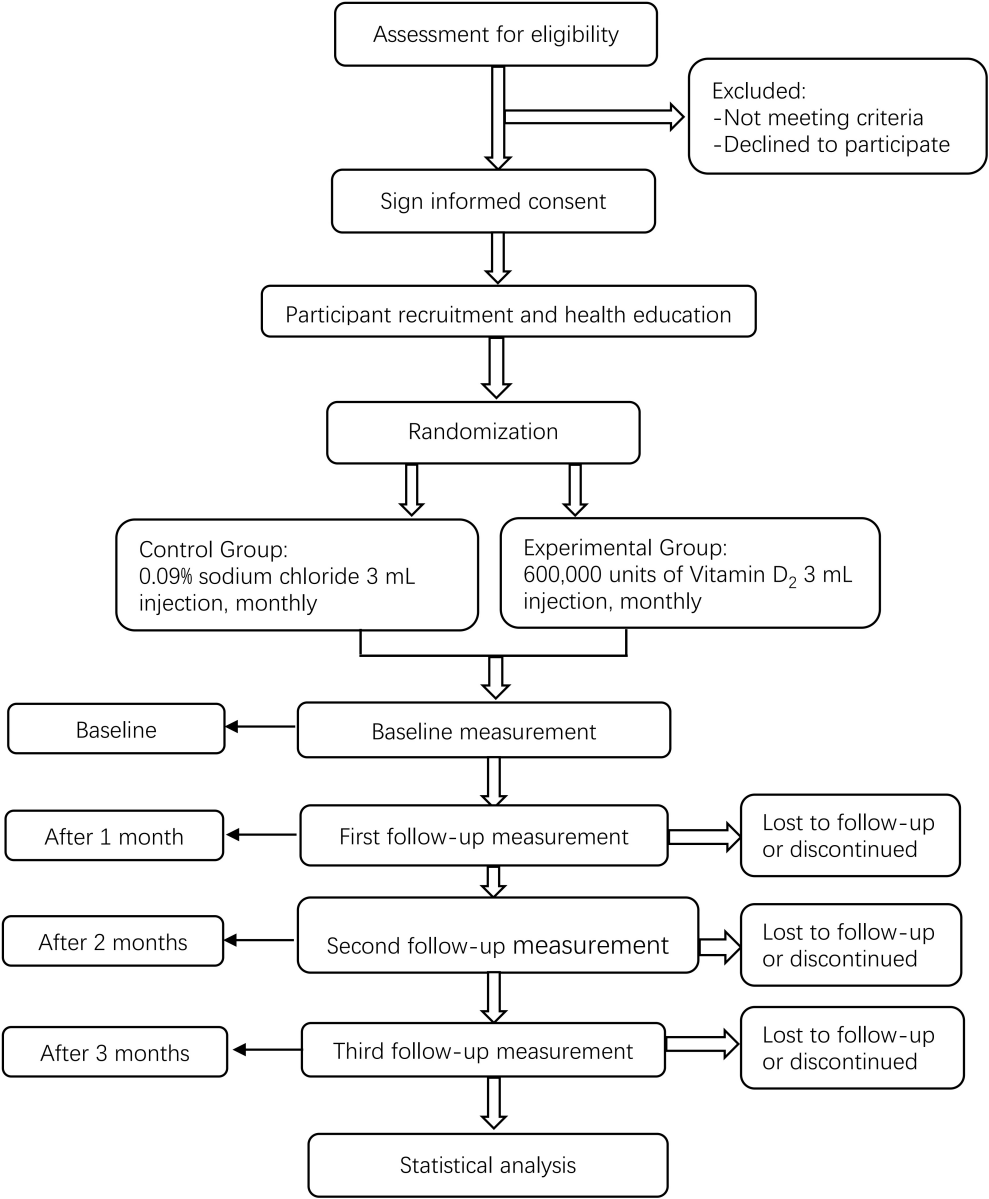
Trial flow chart.

We will conduct a multicenter, prospective, randomized, double-blinded, and placebo-controlled clinical trial. First, participants will be assigned to the HDVD supplementation or placebo control group in a 1:1 block randomization scheme. Next, intramuscular injections will be administered at baseline and 1 and 2 months later. Moreover, we will collect anthropometric measurement data, fasting blood glucose, β-cell function, hemoglobin A1c, and other biochemical data. Lastly, MNSI will be performed at baseline and 3 months later. This study will follow the Comprehensive Reporting Test Standard Guidelines (45). The study flow chart is shown in **Figure 1**.

#### 2.1.1 Study setting

We will recruit participants from four large hospitals: Longyan First Affiliated Hospital of Fujian Medical University, Longyan Traditional Chinese Medicine Affiliated Hospital of Fujian University of Traditional Chinese Medicine, Longyan Boai Hospital, and Longyan Shanghang County Hospital. All participants will provide written informed consent to participate in the study.

#### 2.1.2 Participants

The inclusion and exclusion criteria for the target participants are listed in **Table 1**. All diagnostic criteria will be based on the guidelines and expert consensus of the World Health Organization and China. To recruit as many eligible patients with DSPN as possible, we will distribute recruitment leaflets to outpatients and hospital wards and release recruitment information using the WeChat mobile application (version 8.0.32, Tencent Holdings Limited, Shenzhen, China) and other online forms. Those interested can contact the recruiter directly *via* phone. If a participant experiences a serious adverse event or disease (such as anaphylactic shock or myocardial infarction) during the trial, whether related to the investigational drug or not, the person will be treated immediately and withdrawn from the trial. Moreover, the participants will be allowed to withdraw from the trial at any time.

**Table 1:** Inclusion criteria and exclusion criteria.

Inclusion criteria	Exclusion criteria
1. All participants live permanently in Longyan City, South China (latitude and longitude 115 ° 50 ′ 56 "-117 °44 ′ 15" E, 24 ° 22 ′ 31 "-26 °2 ′ 35" N).	1. Patients without glucose homeostasis such as diabetic ketoacidosis and hypoglycemia.
2. Aged between 20 and 79 years.	2. Patients with hyperthyroidism, hypothyroidism, or parathyroid disease.
3. 25(OH) vitamin D < 30 ng/mL	3. Severe disability or mental illness and pregnant and lactating women.
4. All participants will sign a written informed consent form.	4. Alcoholic or drug abusers, a vegetarian who never eats fish or meat.
5. The diagnosis of type 2 diabetes mellitus will be confirmed according to the diagnostic criteria of the World Health Organization in 1999.	5. Chronic gastrointestinal diseases such as intestinal tuberculosis, ulcerative colitis, or irritable bowel syndrome.
6. The diagnosis of distal symmetric polyneuropathy will be diagnosed according to the Guidelines for the Prevention and Treatment of Type 2 Diabetes Mellitus in China (2020 Edition). Five neurological examinations will be performed, including ankle reflexes, pinprick pain, vibration, pressure, and temperature sensations. If a patient has one clinical symptom (numbness, pain, and paresthesia) and any of the five neurological examinations are abnormal, the patient will be diagnosed with DSPN. Additionally, patients will be diagnosed with DSPN if two of the five neurological examinations are abnormal, without any clinical symptoms, **Figure 2**.	6. The participant has recently taken the following medications or supplements: immunosuppressants or glucocorticoids, such as prednisone at doses >10 mg/day; drugs such as phenytoin sodium, phenobarbital, and rifampicin that may affect the catabolism of vitamin D; vitamin D or active vitamin D, such as calcitriol; and drugs that directly affect nerves (such as chemotherapy drugs, vitamin B6 or Neurobion). 7. Patients with large areas of skin pigmentation or skin diseases. Those who engage in long-term outdoor activities or have tropical tourism plans in the next 3 months. Using sunscreen on the face and hands 8. Hyperphosphatemia and renal rickets.
	9. Blood calcium level >11 mg/dL (2.75 mmol/L), history of urinary tract stones, and sarcoidosis.
	10. Acute stress diseases, such as shortness of breath, fever and diarrhea, severe leukopenia, malignant tumor, liver dysfunction (bilirubin> 1.5 times the upper limit of normal value, alanine aminotransferase> 2 times the upper limit of normal value) or organic diseases, such as decreased renal function eGFR*<20 mL/min/1.73 m2.
	11. Neuropathy caused by other causes, such as cervical and lumbar lesions (nerve root compression, spinal stenosis, cervical and lumbar degeneration), cerebral infarction, Guillain–Barre syndrome, and serious arteriovenous vascular lesions (venous embolism, lymphangitis).
	12. Participants considered unsuitable for the study based on the investigator's judgment.

DSPN, Distal symmetric polyneuropathy; eGFR, estimated glomerular filtration rate.

#### 2.1.3 Sample size estimation

In a previous study of patients with peripheral neuropathy who received vitamin D supplementation for a 1.19-point reduction in MNSI physical examination score with a standard deviation of 1.29 points (46). We assumed a two-sided α error probability of 0.01 and a power of 0.95. We used PASS version 11 (NCSS, LLC. Kaysville, Utah, USA) to estimate the sample size. After estimation, the total sample size was 84 cases, with 42 in each group. Considering that 20% of the participants could be lost to follow-up or refusal of follow-up, we plan to enroll 53 patients per group (total 106 patients).

**Figure 2:**
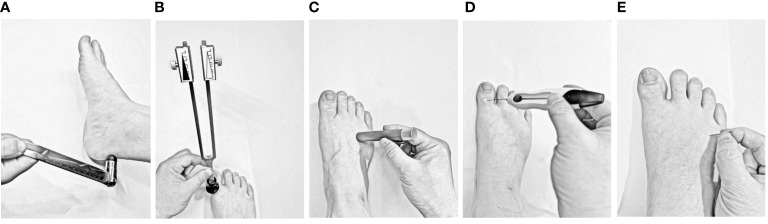
Schematic of five neurological examinations used to diagnose distal symmetric polyneuropathy **(A)** Ankle reflexes: The examiner should gently tap the Achilles tendon on one side of the subject’s foot using a percussion hammer. The ankle reflex is considered absent if the foot cannot be plantarflexed, weakened if the plantar flexion is not significant, and positive if the bilateral ankle reflexes are weakened or absent. **(B)** Vibration sensing: After striking a 128 Hz tuning fork, it is placed on the dorsal side of the big toe of both feet to observe whether the participants can feel the tuning fork’s vibration. Vibrational sensation is positive if the sense of vibration on either side disappears or decreases. **(C)** Temperature sensing: One end of the temperature tester (metal or polyester) is s placed on the skin of the dorsum of the foot (avoiding calluses, ulcers, scars, and necrotic tissue). The result is positive if the participant cannot correctly distinguish the temperature at either end. **(D)** Pressure sensing: A 10 g Semmes–Weinstein monofilament is placed on the dorsal side of the hallux with gradually increasing pressure, repeated four times on each side, eight times in total on both feet, and 1 point is recorded if no pressure is sensed once. The result is considered positive if the total score is ≥ 5 points. **(E)** Pinprick pain sensing: Gently prick the skin of the dorsum of the foot with the tip of a pin without causing skin damage. Participants who cannot feel pain or are hyperresponsive to pain are considered positive.

### 2.2 Randomization, allocation concealment and blinding

All eligible participants will be assigned a unique identification number in the order of enrollment. Randomized assignment of subjects is fundamental to clinical trial design. While performing a simple sequence of random numbers can ensure independence among subjects and avoid potential selection biases, doing so has the potential to lead to an imbalance in the distribution of important covariates across treatment groups. Therefore, we will opt for the block randomization scheme (47). A person independent of the research center will generate a block randomization coding scheme using R software (version 4.1.2, R Foundation for Statistical Computing, Vienna, Austria). Next, all participants will be assigned in a 1:1 ratio to the placebo control or HDVD supplementation groups.

In order to avoid selection bias to the maximum extent possible, we will adopt the method of avoiding leakage of grouping information. The goal is to balance out all known and unknown factors between the two groups, so that differences in outcomes between the groups could ultimately be attributed to the effect of HDVD supplementation. To this end, we are going to use the method of allocation concealment. A staff member who determines the grouping of random sequences will not be involved in the inclusion of subjects. Neither our investigators nor our subjects are aware of the random sequence and the corresponding group during the diagnosis of DSPN, during the eligibility evaluation stage such as reviewing whether patients met the inclusion and exclusion criteria, and during the stage of soliciting patients’ willingness to participate in the trial.

Each protocol will be placed in an opaque envelope, marked with the code, and sealed. In order to reduce information bias, including measurement bias and implementation bias, we will double-blind the trial for the investigator and participants. Once the participants are enrolled, a third person, independent of the participant and investigator, will open the envelopes individually, and the executor will perform the corresponding injection per the envelope protocol.

### 2.3 Intervention

All participants will receive diabetes health education and follow-up. Medical nutrition therapy will be recommended for every patient with diabetes, and they will be advised to avoid overeating and undereating, which causes oxidative stress due to glucose fluctuations, exacerbating peripheral nerve damage. Moreover, self-monitoring of blood glucose will be recommended for every participant. The optimal blood glucose control target will be 4.4 mmol/L < fasting blood glucose < 7.0 mmol/L, 2-h postprandial blood glucose < 10.0 mmol/L, and avoid hypoglycemia. Next, exercise therapy, avoidance of foot injuries, and wearing white socks frequently will be recommended for every patient with diabetes to detect minor local injuries early.

After randomization, all participants will receive an intramuscular injection of the trial drug (vitamin D_2_ 600,000 units, 3 mL, monthly for 3 months) or placebo (0.09% sodium chloride 0.0027 g, 3 mL, monthly for 3 months) into the gluteus maximus. Vitamin D_2_ is produced by Jiangxi Gannan Haixin Pharmaceutical Co. Ltd. (Ganzhou, Jiangxi, China), and sodium chloride by Hubei Xinghua Pharmaceutical Co. Ltd. (Wuhan, Hubei, China). Each injection will be packaged in an opaque sealed package with an instruction leaflet and label. Drug dispensing will be performed by a third person independent of the implementer and participant. It is critical that all participants remain in the follow-up cohort as long as possible, and the efforts of all investigators and related personnel will improve participant adherence to the intervention and follow-up schedule. If the participant refuses to continue the follow-up, the investigator will determine the reason and encourage the participant to continue the intervention and follow-up.

### 2.4 Outcome assessment

#### 2.4.1 Primary and secondary outcomes

The primary outcome measure is the change in the mean MNSI score at baseline and 3 months post-intervention, reflecting the peripheral neuropathy status of the participant.

The secondary outcome measures include 1. changes in vitamin D level, including 25(OH) D_2_, 25(OH)D_3_, and 25(OH)D; 2. changes in fasting blood glucose, 2-h postprandial blood glucose, and hemoglobin A1c; 3. changes in fasting C-peptide and 2-h postprandial C-peptide; 4. changes in homeostasis model 2 for assessing insulin resistance, insulin sensitivity, and β-cell function (HOMA2-IR, HOMA2-%S, and HOMA2-%B, respectively) by HOMA2 software (version 2.2) available from the Oxford Centre for Diabetes, Endocrinology, and Metabolism (48).; 5. changes in systolic blood and diastolic blood pressure; 6. changes in triglyceride, total cholesterol, high-density lipoprotein cholesterol, and low-density lipoprotein cholesterol; and 7. changes in the maximum bimanual grip strength and quality of life score.

#### 2.4.2 Demographic and anthropometric data

Baseline data will be collected at enrollment for all participants. The demographics will include name, age, sex, race, residence, occupation, marital status, and education. Anthropometric measurements will include height, weight, body mass index, waist circumference, and hip circumference. Additionally, we will measure vital signs, such as body temperature, heart rate, and blood pressure, simultaneously.

#### 2.4.3 Clinical data collection and follow-up

Venous blood samples will be collected from participants at baseline and designated visit points for biochemical data, such as lipid, glucose, and uric acid. The method of venous blood sampling is as follows. First, the participants will fast for 10–12 h, and a qualified nurse will collect blood samples at 6:30–8:30 am. Next, the blood samples will be tested for 25(OH)D_2_, 25(OH)D_3_, and 25(OH)D levels using high-performance liquid chromatography-tandem mass spectrometry (LC-MS/MS) at the Fuzhou King Med Clinical Laboratory (Fuzhou, Fujian, China). The executive assistants involved in data collection and evaluation will be blinded to participants’ groupings. All clinical data collection processes are presented in **Table 2**.

**Table 2:** Participant flow of enrolment, intervention, and outcome measurements.

		PS	M0	M1	M2	M3
Enrolment	Recruitment	√				
	Eligibility Screening	√				
	Signed written informed consent	√				
	Allocation		√			
Intervention	Health education, medical nutrition therapy, exercise therapy, self-monitoring of blood glucose, and disease follow-up		√	√	√	√
	High-dose vitamin D_2_ /placebo injection		√	√	√	
Outcomes	Demographic characteristics and basic information (sex, age, marital status, place of residence, and education level)		√			
	Personal life status (smoking status, drinking status, beverage intake status, sunshine reception, living alone, and menstrual status for females)		√			
	Duration of diabetes, strict diet control, and moderate aerobic exercise for >150 min per week		√			
	Number of self-monitoring of blood glucose per week and occurrence of hypoglycemia		√			
	Comorbidities (hypertension, hyperuricemia, obesity, osteoporosis, and nonalcoholic fatty liver disease)		√			
	Other diabetic complications (diabetic retinopathy, diabetic nephropathy, diabetic foot ulcer, coronary atherosclerotic heart disease, cerebrovascular disease, and peripheral vascular disease)		√			
	Anthropometric measurements (height, weight, body mass index, waist, and hip circumference) and blood pressure (systolic and diastolic blood pressure)		√			√
	Metabolic parameters (FBG, 2hBG, HbA1C, FCP, 2hCP, triglycerides, total cholesterol, HDL-C, LDL-C, uric acid, HOMA2-IR/%S/%B)		√			√
	Intima-media thickness of carotid and dorsalis pedis artery by ultrasound, fundus examination, bone mineral density		√			√
	25-(OH) Vitamin D_2_, 25-(OH) Vitamin D_3_, and 25-(OH) Vitamin D tested by LC-MS/MS method		√			√
	Maximum bimanual grip strength measured by a dynamometer		√			√
	Short Form -12 Quality of Life Questionnaire		√			√
	MNSI score		√			√

PS, prior to study; M0, baseline assessment; M1, 1 month from baseline; M2, 2 months from baseline; M3, 3 months from baseline; HOMA2-IR/%S/%B; homeostasis model assessment index 2 for assessing insulin resistance/insulin sensitivity/function of islet β-cells; LC-MS/MS, liquid chromatography-tandem mass spectrometry; ^*^MNSI, Michigan Neuropathy Screening Instrument; FBG, fasting blood glucose; HbA1c, hemoglobin A1c; 2hBG, 2-h postprandial blood glucose; FCP, fasting C-peptide; 2hCP, 2-h postprandial C-peptide; HDL-C, high-density lipoprotein cholesterol; LDL-C, low-density lipoprotein cholesterol.

#### 2.4.4 Safety assessment and adverse events reporting

We will evaluate liver function, albumin, renal function, calcium, phosphorus, potassium, sodium, chloride, and other biochemical parameters at baseline and post-intervention to evaluate the safety of the HDVD. All adverse events reported during the study will be closely monitored and recorded. In this trial, an adverse event is any unfavorable or unexpected occurrence related to the intervention. The investigator will ask the participants questions regarding adverse reactions. Participants will be called on days 1, 3, and 7 after each injection to determine any adverse events (**Table 3**). Moreover, participants will be asked to self-report any adverse events at any time. All adverse events will be professionally addressed, closely monitored by a physician, and treated as necessary. Serious adverse events will be reported to the study director—responsible for managing adverse reaction reports—within 1 h. Additionally, related serious adverse events will be reported to the Ethics Committee of Longyan First Affiliated Hospital of Fujian Medical University. The Ethics Committee is independent of the investigators, and the investigators are not members of the Ethics Committee.

**Table 3:** Safety assessment and adverse event follow-up of participant.

	M0	M1	M2	M3
Liver function: alanine aminotransferase, aspartate aminotransferase, total bilirubin, albumin, globulin	√			√
Renal function: creatinine, blood urea nitrogen, and eGFR calculated by the CKD-EPI equation	√			√
Creatine kinase (CK), creatine kinase myocardial band (CK-MB), lactate dehydrogenase (LDH), alkaline phosphatase (ALP)	√			√
Electrolytes: calcium (Ca), phosphorus(P), potassium(K), magnesium (Mg), sodium (Na), chloride (Cl)	√			√
Closely follow up on the physical health after injection, and receive consultation and feedback at any time within 24 h	√	√	√	√
Did the participant experience any adverse reactions after the injection (whether or not determined to be related to this injection)	√	√	√	√
Digestive adverse reactions: decreased appetite, constipation or diarrhea, abdominal pain, nausea, or vomiting	√	√	√	√
Skin adverse reactions: pruritus, allergic dermatitis, rash, swelling, and pain at the injection site	√	√	√	√
Nervous system adverse reactions: metallic sensation in the mouth, persistent headache, convulsions, and insomnia	√	√	√	√
Urinary adverse reactions: cloudy urine, nocturnal polyuria, and hematuria	√	√	√	√
Skeletal system adverse reactions: bone pain and myalgia	√	√	√	√
Respiratory and circulatory system: shortness of breath, palpitations, hypertension, shock, hypotension, and chest pain	√	√	√	√
Hematologic and endocrine adverse reactions: rapid weight loss or gain, fever, and fear of cold	√	√	√	√
Other adverse reactions: fatigue, asthenia, dry mouth, and fall	√	√	√	√
Record treatment for adverse reactions (only for participants with adverse events)	√	√	√	√

M0, baseline assessment; M1, 1 month from baseline; M2, 2 months from baseline; M3, 3 months from baseline; eGFR, estimated glomerular filtration rate; CKD-EPI, the Chronic Kidney Disease Epidemiology Collaboration equation.

### 2.5 Data management

After enrollment, all participants will be given a unique identification number, which will substitute participants’ names in the database to protect their privacy. All data will be entered using the EpiData Software (Version 3.1, Denmark), and two data entry clerks will enter the case report form into the same database and cross-check to ensure the accuracy of the data. Finally, all written materials and encrypted electronic files containing the study’s data will be stored and backed by the study director. These documents will be retained for > 3 years after this study.

### 2.6 Data analysis

Any participant who completes the randomization will be included in the intention-to-treat set for data analysis. In cases when random or unforeseeable missing data occur, the missing data will be processed by multiple imputation method and sensitivity analysis will be carried out (49). The safety set which contains at least one safety assessment and adverse event report will be used to analyze the safety of HDVD supplementation. Quantile-quantile plot will be used to determine if the data conforms to a normal distribution. For descriptive analysis, the results of continuous variables (age and body mass index, among others) that conform to normal distribution will be expressed as mean ± standard deviation, and those that do not conform to normal distribution will be expressed as median and interquartile range. Categorical variables (sex and smoking status) will be expressed as frequencies and percentages.

Baseline data will be compared between the HDVD supplementation and placebo control groups. In HDVD supplementation or placebo control group, a paired sample t-test will be used for post-intervention versus pre-intervention comparisons. The difference value of MNSI before and after intervention will be performed by independent sample t-test test to compare the difference between the two groups. If the data does not conform to normal distribution, the Mann-Whitney U test will be used for analysis. The chi-square test will be used for categorical variables.

Furthermore, we will use the MNSI score before intervention as a covariate, and the F value will be calculated using analysis of covariance (ANCOVA) to analyze the change in the MNSI score after HDVD supplementation. Finally, if the number of adverse events occurring during the study is sufficient, the chi-square test or Fisher’s exact probability method will be used to compare differences in adverse events between both groups.

All data will be analyzed using the R software (version 4.1.2; R Foundation for Statistical Computing, Vienna, Austria). Additionally, all statistical analyses will be performed using a two-sided test, considering P < 0.05 statistically significant.

### 2.7 Data monitoring

This study will be supervised by the Ethics Committee of the Longyan First Affiliated Hospital of Fujian Medical University, whose members have no conflicts of interest with this study. The study director will have access to all results and decide when to terminate the study based on its progress. Additionally, the study director will grant other members of the study’s team the right to disseminate the trial results by publishing papers.

### 2.8 Special instructions

During the study, participants will be told to avoid high-fat and high-carbohydrate diets inappropriate for T2DM patients. Moreover, participants will be informed to avoid excessive sun exposure and consumption of salmon, mushrooms, and other foods that may influence vitamin D levels. Furthermore, participants will be asked to spend 20 min daily in the sun outdoors as usual. Next, participants will be notified 3 days before the next injection and follow-up to avoid being unable to participate in the injection in time due to a busy schedule or forgetting the time. Moreover, if the participants cannot visit the hospital on time on weekdays, executive assistants will use overtime to inject and follow them up as necessary on a case-by-case basis. Finally, we will provide a 24-h telephone consultation service and other measures to ensure the best attendance and avoid losses to follow-up.

## 3 Discussion

DSPN can induce diabetic foot ulcer and amputation, increasing the social burden and consuming medical resources. The United States spends > $10 billion yearly to treat DSPN. Early identification and treatment of DSPN can save at least 80% of medical costs (50–52). Therefore, preventing the further development of DSPN has attracted our attention.

Vitamin D deficiency is a growing global health problem, even in countries like the United Arab Emirates, which receives plenty of sunshine yearly (18). This implies that the vitamin D produced by the sun’s ultraviolet rays is insufficient. A recent meta-analysis showed that vitamin D deficiency in T2DM increased the risk of DSPN by 1.22 times (53). This evidence highlights an early treatment window for patients with T2DM and the possibility that vitamin D supplementation may prevent the development of DSPN. A previous retrospective study found that vitamin D levels in patients with T2DM and DSPN were one-third lower than those without DSPN, suggesting that vitamin D may affect DSPN progression (18). Furthermore, clinical studies have shown that HDVD supplementation can significantly reduce the pain score of DSPN, reduce demyelination, improve axonal regeneration, and even improve the quality of life of patients with DSPN (33, 34, 54).

Notably, patients with vitamin D deficiency may not rely solely on increased sun exposure and dietary supplements; therefore, additional vitamin D supplements are needed. Supplementation is achieved mainly by oral administration and intramuscular injection of vitamin D pharmaceutical preparations. However, recovery from neuropathy is slow, and most patients have difficulty maintaining a daily low dose of vitamin D supplements. Moreover, it also takes longer to achieve vitamin D sufficiency with low-dose supplementation.

Since low-dose vitamin D supplementation cannot quickly correct this deficiency, some scientists have begun to try HDVD supplementation programs. A clinical study conducted by Masood et al. (55) with a single dose of 600000 units of vitamin D showed that it took only 2 months for 70% of people with vitamin D deficiency to achieve an adequate status, indicating that high-dose vitamin D supplementation can correct vitamin D deficiency quickly. This means the high-dose regimen could reduce the time traditionally required to reach the target. Additionally, a previous study by Xu et al. (56) demonstrated that a single injection of 600000 units of vitamin D increased 25(OH)D level by 10.3 ng/mL over 12 weeks, indicating that HDVD injection therapy is effective and durable in raising vitamin D concentrations. A clinical study by Diamond et al. (35) showed that a regimen of 600000 units of vitamin D injected intramuscularly once yearly increased participants’ vitamin D levels by an average of 128%, and all participants’ vitamin D levels were normalized. Furthermore, a bioavailability study by Cipriani et al. (57) revealed that vitamin D levels peaked on day 120 after a single intramuscular dose of 600000 units of vitamin D, and the conversion to 1,25 (OH)_2_D was more stable and less volatile after intramuscular administration than after oral administration. Overall, the studies described above suggest that intramuscular HDVD is a more effective method of vitamin D supplementation than daily oral low-dose vitamin D supplementation. Moreover, the HDVD regimen has better compliance owing to fewer supplements, no need to take medication daily, and less forgetting or omission. However, few studies have investigated whether an HDVD can improve peripheral neuropathy. Our study aims to determine the efficacy of HDVD in T2DM patients with DSPN.

To make our study feasible, our study plan will exclude patients with severe renal insufficiency. Because 1α-hydroxylase is tightly regulated in the body, safe dose range for vitamin D supplementation is wide and there is no excess of 1,25-(OH)_2_D that is formed in the body. Previous studies have shown that poisoning is only possible when the 25-hydroxyvitamin D levels is greater than 224 ng/mL (560nmol/L) (17, 58, 59). A prospective randomized controlled study by Mehta et al. showed significant improvement in neuropathy in patients with DSPN who received HDVD supplementation for 6 months. The study included patients with chronic kidney disease (CKD) stages 1-4 and excluded patients with CKD stage 5 (eGFR <15 mL/min/1.73 m^2^). The authors showed that 25-hydroxyvitamin D levels were significantly increased 6 months after the HDVD supplementation (before: 18.91 ± 5.3 ng/mL vs. after: 53.71 ± 7.8 ng/mL, *p*<0.05); however, serum creatinine level was not increased (before: 1.99 ± 0.9 mg/dL vs. after: 1.82 ± 1.8 mg/dL,*p*>0.05). This study provides strong evidence that HDVD supplementation does not worsen renal function in DSPN patients with an eGFR greater than 15 mL/min/1.73 m^2^ (60). It is well known that kidney function gradually declines with age; however, a clinical study of monthly intramuscular injection of HDVD in very elderly people with an average age of 89.6 years has attracted our attention. The results of this study showed no further decline in renal function after intervention (creatinine level, before: 97.38 ± 18.08 mg/dL vs. after: 101.12 ± 20.39 umol/L, *p*>0.05), and there was no significant change in blood calcium levels (before: 2.30 ± 0.14 mg/dL vs. after:2.34 ± 0.11 mmol/L, *p*>0.05) (61). Our study will also collect data on changes in renal function, blood calcium after HDVD.

Many researchers have explored whether increasing the dose of vitamin D supplementation will cause side effects or adverse reactions. In a study by Binkley et al. (37) administering 50000 units of vitamin D monthly to people aged > 65 years did not cause any toxicity or side effects. Another clinical study by Bian et al. (61) in people aged > 80 years showed that supplementation with 600000 units of vitamin D monthly for more than half a year might be safe. However, the sample size used in these studies was small. Therefore, we will use a monthly regimen of high-dose intramuscular vitamin D injections closely observed by the implementer to achieve better compliance and an earlier time to target. Additionally, our study will address the safety of monthly HDVD supplementation in individuals with insufficient vitamin D levels.

Although vitamins D_2_ and D_3_ have the same efficacy in exerting physiological effects, previous studies have suggested that vitamin D_2_ supplementation may affect vitamin D_3_ levels (56). This may be related to increased CYP24A1 enzyme activity in the liver, promoting the conversion of 25(OH)D_3_ to 24,25-(OH)_2_D_3_, thereby inactivating it (57, 62). Additionally, 25(OH)D_2_ and 25 (OH)D_3_ compete for the binding to VDBP (63, 64). Notably, traditional immunoassays for vitamin D do not distinguish between 25(OH)D_2_ and 25(OH)D_3_, which may underestimate 25(OH)D_2_ levels. Testing for vitamin D_2_ can rule out the effects of sunlight (65). Therefore, we will use the gold standard LC-MS/MS method to distinguish between 25(OH)D_2_ and 25 (OH)D_3_ levels.

The strength of this study design is that it will determine whether an improvement in neuropathy accompanies an increase in vitamin D concentration due to HDVD supplementation. Notably, DSPN is associated with muscle atrophy; thus, decreasing the quality of life. Although previous studies suggested that vitamin D supplementation may improve muscle strength (52, 54, 66), our study will explore the effects of HDVD from multiple dimensions, such as quality of life and muscle strength. We will use the Short Form-12 (SF-12) questionnaire, comprising eight domains, to assess the quality of life. The SF-12 has satisfactory validity and reliability, showing good internal consistency in the psychological (Cronbach’s α=0.83) and physical component summaries (Cronbach’s α=0.81) (67). Moreover, we will measure the maximum grip strength of both hands before and 3 months post-intervention to assess changes in muscle strength.

This study design has some limitations. First, the trial will be conducted only in China’s population with T2DM and DSPN. Therefore, the results may not be directly applicable to other ethnic groups. Second, the latitudes of southern China will be selected for this study; therefore, the results may not be directly applicable to other latitudes. Nevertheless, the study can provide certain evidence that may require further verification in other regional or international multicenter studies. Third, owing to the difficulty in recruiting patients with DSPN, it will be difficult to maintain a balance between the groups. However, we will further correct factors, such as age, sex, and body mass index, through stratified analysis and by applying the analysis of covariance statistical strategy as far as possible.

In conclusion, this study will provide clinical evidence demonstrating the efficacy and safety of post-treatment HDVD supplementation for DSPN. Additionally, if HDVD supplementation can improve neuropathy, this study will provide new directions for future pharmacological research and clinical management of diabetic neuropathy.

## Data availability statement

The original contributions presented in the study are included in the article/supplementary material. Further inquiries can be directed to the corresponding author.

## Ethics statement

The studies involving human participants were reviewed and approved by the Ethics Committee of Longyan First Affiliated Hospital of Fujian Medical University (approval number: LYREC2022-013-01). The patients/participants provided their written informed consent to participate in this study.

## Author contributions

TC, XX, PW, and MT participated in the study design. XX, PW, and MT supervised the study.TC prepared the first of the manuscript. TC and SL conducted the statistical strategy. LH performed sample size estimation. LH, XL, JC, SW, YZ, YL, LX, YQ, LQ, and YX edited and revised the manuscript. PW reviewed the methodology and the entire contents of the manuscript. All authors contributed to the article and approved the submitted version.
